# Origin and cell type specificity of mitochondrial DNA mutations in *C9ORF72* ALS-FTLD human brain organoids

**DOI:** 10.1126/sciadv.adr0690

**Published:** 2025-03-07

**Authors:** Yu Nie, Kornélia Szebényi, Lea M. D. Wenger, András Lakatos, Patrick F. Chinnery

**Affiliations:** ^1^ Department of Clinical Neurosciences, School of Clinical Medicine, University of Cambridge, Cambridge Biomedical Campus, Cambridge, UK.; ^2^ Medical Research Council Mitochondrial Biology Unit, University of Cambridge, Cambridge Biomedical Campus, Cambridge, UK.

## Abstract

Amyotrophic lateral sclerosis (ALS) and frontotemporal lobar degeneration (FTLD) are primarily genetic in ~20% of patients. Mutations in *C9ORF72* are the most frequent cause, but it is not understood why there is notable regional pathology. An increased burden of mitochondrial DNA (mtDNA) mutations in ALS-FTLD brains implicates mitochondrial mechanisms; however, it remains unclear how and when these mutations arise. To address this, we generated cerebral organoids derived from human-induced pluripotent stem cells (hiPSCs) of patients with ALS-FTLD harboring the *C9ORF72* hexanucleotide repeat expansion alongside CRISPR-corrected isogenic and healthy controls. Here, we show a higher mtDNA single-nucleotide variant (mtSNV) burden in astroglia derived from *C9ORF72*-mutant organoids, with some de novo mtSNVs likely due to the *C9ORF72* repeat and others evading selection to reach higher heteroplasmy levels. Thus, the functional consequences of the regional accumulation of mtSNVs in *C9ORF72* ALS-FTLD brains are likely to manifest through astroglial mitochondrial dysfunction.

## Teaser

Cells in ALS-FTLD brain organoids show distinct mtDNA mutation landscape indicating mitochondrial dysfunction.

## INTRODUCTION

Frontotemporal lobar degeneration (FTLD) is the second most common cause of early-onset dementia and primarily affects the frontal and temporal cerebral lobes, lying on a spectrum of neurodegenerative diseases that encompasses amyotrophic lateral sclerosis (ALS). The molecular pathophysiology involves aberrant protein processing leading to Tau, TAR DNA-binding protein 43 (TDP-43), fused in sarcoma (FUS), or ubiquitin inclusions with subsequent neuronal dysfunction and cell death (*1*). High-penetrance inherited pathogenic mutations in *C9ORF72*, *GRN*, *MAPT*, *TARDBP*, and *FUS* have been implicated in ~20% of patients with FTLD and ALS-FTLD (*2*), with the GGGGCC hexanucleotide repeat expansion (HRE) in *C9ORF72* being the most common (*3*). Although causal mutations in *C9ORF72* are present in every cell, the associated neuropathology is notably focal and corresponds to the clinical presentation. The specific reasons for the selective cell and regional pathology are not known.

Regional differences in the burden of mitochondrial DNA (mtDNA) mutations have been described in sporadic and genetic forms of FTLD and ALS-FTLD (*CHCHD10* and *C9ORF72*) (*4*–*6*), raising the possibility that they contribute to the focal pathology through an effect on oxidative phosphorylation. Previous studies focused on autopsy samples at an advanced stage in the disease and analyzed bulk tissue samples. Since these strategies do not allow elucidations of the clonal origin and cell type specificity of mtDNA mutations, it remains unclear whether the different mutational burden merely reflects a change in the underlying cell composition.

Human-induced pluripotent stem cell (hiPSC)–derived cerebral organoids recapitulate brain cell type diversity and interactions, showing great promise for exploring dynamic pathobiological processes in a longitudinal and cell type–specific manner (*7*). Therefore, to determine when and how mtDNA mutations contribute to the pathogenesis of ALS-FTLD, we performed single-cell mtDNA sequencing analysis on human cerebral organoids derived from *C9ORF72*-mutant hiPSC lines from patients with ALS-FTLD and compared to isogenic, mutation-corrected healthy control lines (*8*). We show that most mtDNA mutations preferentially accumulate in astroglia by evading selection pressures during cortical development and through new mutation events. The mutations reach levels predicted to compromise mitochondrial function at a single-cell level and are not buffered by an increase in mtDNA content.

## RESULTS

### Isolation of astroglia and neurons from human cerebral organoids

**Fig. 1.**
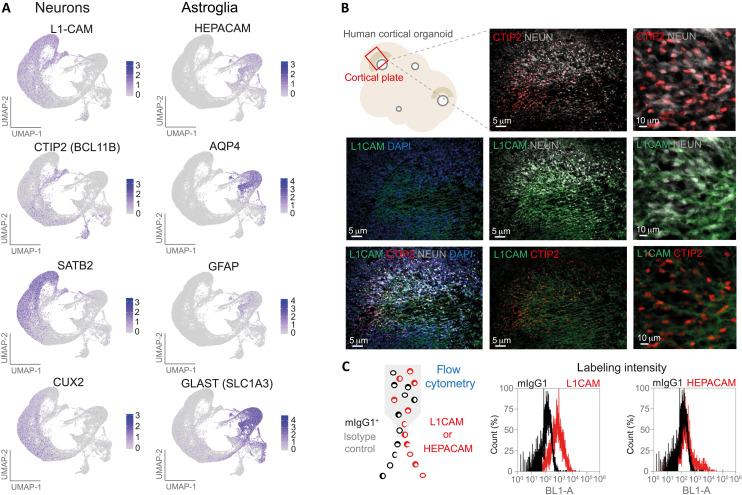
Validation and gating of *HEPACAM*-expressing astroglia and *L1CAM*-expressing neurons in dissociated organoids using FACS. (**A**) Feature plots demonstrating the expression of astroglia and neuronal subtype markers in cortical organoids. Plots represent merged data of human *C9ORF72*-mutant cortical organoids and controls from the study of Szebényi *et al.* (*8*). Color scale represents normalized gene expression levels per cell (blue, high; gray, low). (**B**) Schematic illustration of organoid cortical plates and representative images of L1CAM immunoreactivity overlapping with neuronal nuclear antigen (NeuN) and other neuronal markers in human *C9ORF72*-mutant organoids (*n* = 3, independent organoids). (**C**) Schematic illustration of fluorescence-assisted flow cytometry for the verification of L1CAM and HEPACAM labeling intensities against isotype control antibodies [mouse immunoglobulin G1 (mIgG1)]. DAPI, 4′,6-diamidino-2-phenylindole.

We initially studied four hiPSC lines: two from patients with ALS-FTLD with *C9ORF72*-mutations (als-ftld.a and als-ftld.b); one CRISPR-Cas9–mediated corrected isogenic control after excision of the hexanucleotide repeat in the *C9ORF72* gene (ctrl.a); and one healthy population control as a reference (ctrl.b). Organoids were developed from a *C9ORF72*-mutant hiPSC line and the corresponding isogenic control. After 133 days of culture, the organoids showed similar cell type diversity and cytoarchitecture. Feature plots from single-cell RNA sequencing (scRNA-seq) experiments supported the expression of hepatic and glial cell adhesion molecule (*HEPACAM*), aquaporin 4 (*AQP4*), glial fibrillary acidic protein (*GFAP*), and glutamate aspartate transporter (*GLAST*) or solute carrier family 1 Member 3 (*SLC1A3*) in astroglia; cortical neuronal expression of L1 cell adhesion molecule (*L1CAM*) accompanied by COUP-TF interacting protein 2 (*CTIP2*) or B-cell lymphoma/leukemia 11B (*BCL11B*) in deep layer cortical neurons and special AT-rich sequence-binding protein- 2 (*SATB2*) and homeobox protein cut-like 2 (*CUX2*) in upper layer cortical neurons (Fig. 1A) (*8*). Fluorescence-activated cell sort (FACS) gating based on the immunoreactivity for astroglia marker (*HEPACAM*) and neuronal marker (*L1CAM*) allowed the isolation of astroglia and neurons from dissociated 133-day-old organoids (Fig. 1, B and C, and fig. S1).

### Single-cell mtDNA mutation burden and heteroplasmy levels

**Fig. 2.**
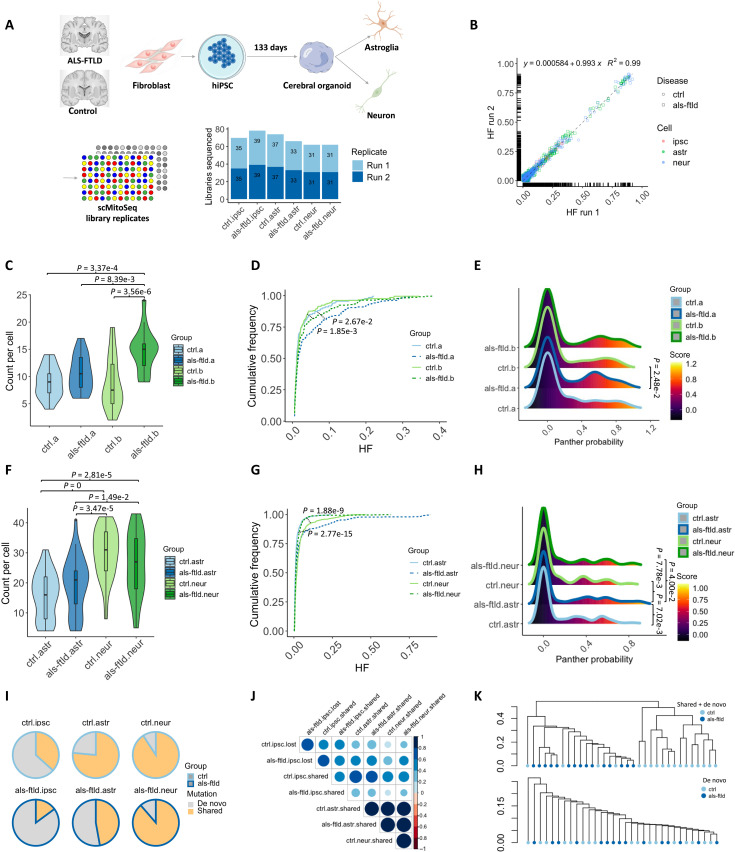
mtSNVs detected in hiPSCs and 133-day-old cerebral organoid-derived astroglia and neurons. (**A**) Schematic diagram showing the workflow: the generation of cerebral organoids from both *C9ORF72*-mutant and isogenic healthy control hiPSCs, cell disassociation, cell type marking, single-cell sorting, and mtDNA sequencing in replicates. (**B**) Correlation of HF of mtSNVs detected in NGS technical replicates (run 1 and run 2). (**C** to **E**) Count per cell [one-way analysis of variance (ANOVA) with post hoc Tukey test: ctrl.a versus als-ftld.b, *P* = 3.37 × 10^−4^; ctrl.b versus als-ftld.b, *P* = 3.56 × 10^−6^; als-ftld.a versus als-ftld.b, *P* = 8.39 × 10^−3^], cumulative frequency (Kolmogorov-Smirnov test: ctrl.a versus als-ftld.a, *P* = 2.67 × 10^−2^; ctrl.b versus als-ftld.b, *P* = 1.85 × 10^−3^), and Panther pathogenicity score (Wilcoxon test: als-ftld.a versus ctrl.b, *P* = 2.48 × 10^−2^) of mtSNVs detected in two *C9ORF72*-mutant and two control hiPSCs. (**F** to **H**) Count per cell (one-way ANOVA with post hoc Tukey test: als-ftld.astr versus ctrl.neur, *P* = 3.47 × 10^−5^; als-ftld.astr versus als-ftld.neur, *P* = 1.49 × 10^−2^; ctrl.astr versus ctrl.neur, *P* = 0; ctrl.astr versus als-ftld.neur, *P* = 2.81 × 10^−5^), cumulative frequency (Kolmogorov-Smirnov test: ctrl.astr versus als-ftld.astr, *P* = 2.77 × 10^−15^; ctrl.neur versus als-ftld.neur, *P* = 1.88 × 10^−9^), and Panther pathogenicity score (Wilcoxon test: ctrl.astr versus als-ftld.astr, *P* = 7.02 × 10^−3^) of mtSNVs detected in astroglia and neurons derived from a *C9ORF72*-mutant case and its isogenic control. (**I**) Percentage of shared and de novo mtSNVs detected in each *C9ORF72*-mutant and control cell type. (**J**) Correlation matrix of trinucleotide mutational signatures of mtSNVs detected in hiPSCs but not organoid cells (lost) or shared between hiPSCs and organoid cells (shared). (**K**) Hierarchical clustering analysis of hiPSCs based on all mtSNVs (shared and de novo) or solely de novo mtSNVs detected in each cell. ipsc, hiPSCs; astr, astroglia; neur, neurons; ctrl, control; als-ftld, *C9ORF72* mutant. scMitoSeq, single cell mitochondrial genome sequencing.

Two hundred and six single cells were sequenced in duplicate (referred to as run 1 and run 2) from four hiPSC lines and two organoids (total cells sequenced = 74 from hiPSCs, 62 from organoid neurons, and 70 from organoid astroglia; Fig. 2A). The mean mtDNA sequencing depth was 2515 (range, 22 to 17,532) for run 1 and 2681 (range, 32 to 18,791) for run 2. To minimize sequencing artifacts, mtDNA single-nucleotide variants (mtSNVs) with heteroplasmy fraction (HF) exceeding 0.005 (0.5%) in both run 1 and run 2 were identified using a validated bioinformatic approach (*9*). A strong correlation was observed in the HF across both runs (linear regression model: *R*^2^ = 0.99; Fig. 2B). Subsequent analyses were therefore restricted to mtSNVs in both runs with HF > 0.005 where we had a very high confidence that the variants were present in the original DNA samples (*10*).

A mean of 8 (ctrl.a) and 8 (ctrl.b) mtSNVs were detected per cell in the control hiPSCs and a mean of 10 per cell (als-ftld.a) and 14 per cell (als-ftld.b) in the *C9ORF72*-mutant hiPSCs (ctrl.a versus als-ftld.b, ctrl.b versus als-ftld.b, and als-ftld.a versus als-ftld.b, *P* < 0.05; Fig. 2C). The *C9ORF72*-mutant hiPSCs had a higher cumulative HF than controls in both biological replicates (ctrl.a versus als-ftld.a and ctrl.b versus als-ftld.b, *P* < 0.05; Fig. 2D). mtSNVs detected in *C9ORF72*-mutant hiPSCs had a higher mean pathogenicity probability than control hiPSCs based on the Panther score (based on position-specific evolutionary conservation; als-ftld.a versus ctrl.b, *P* < 0.05; Fig. 2E) and the MutPred score (based on whether a variant introduces an amino acid substitution; als-ftld.a versus ctrl.b, *P* < 0.05; fig. S2).

A mean of 15 mtSNVs were detected per cell in astroglia and 29 mtSNVs per cell in neurons isolated from the mutation-corrected isogenic control organoids (ctrl.a) aged 133 days. A mean of 19 mtSNVs were detected per cell in astroglia and 26 mtSNVs per cell in neurons isolated from the *C9ORF72*-mutant organoids (als-ftld.a) of the same age (als-ftld.astr versus ctrl.neur, als-ftld.astr versus als-ftld.neur, ctrl.astr versus ctrl.neur, and ctrl.astr versus als-ftld.neur, *P* < 0.05; Fig. 2F). Astroglia isolated from *C9ORF72*-mutant organoids had a higher HF than astroglia in isogenic control organoids. In comparison, neurons isolated from *C9ORF72*-mutant organoids had a lower HF than control, although to a smaller extent (ctrl.astr versus als-ftld.astr and ctrl.neur versus als-ftld.neur, *P* < 0.05; Fig. 2G). This was independently confirmed by bulk sequencing (fig. S3). mtDNA mutations in *C9ORF72*-mutant organoid-derived astroglia were more likely to be pathogenic than those in control organoid astroglia based on Panther and MutPred score predictions (ctrl.astr versus als-ftld.astr, *P* < 0.05; Fig. 2H; ctrl.astr versus als-ftld.astr, *P* < 0.05; fig. S2). A similar trend was also previously observed following deep mtDNA sequencing of FTLD human postmortem brains tissue (*6*).

### Origin of mtSNVs

Next, we turned our attention to the likely origin of mtSNVs. A total of 36.64% of mtSNVs per cell were overlapping between control hiPSCs and organoid-derived cells (shared mtSNVs), 63.35% were present only in hiPSCs (lost mtSNVs), 23.51% were present only in the organoid-derived astroglia, and 9.19% were present only in the organoid-derived neurons (likely de novo variants). In *C9ORF72* mutants, 14.90% of mtSNVs per cell were overlapping between control hiPSCs and organoid-derived cells (shared mtSNVs), 85.09% were present only in hiPSCs (lost mtSNVs), 52.83% were present only in the organoid-derived astroglia, and 11.38% were present only in the organoid-derived neurons (likely de novo variants) (Fig. 2I). The mutational signature of the shared and lost mtSNVs was similar, consistent with a similar molecular origin (Fig. 2J). On the basis of hierarchical cluster analysis of the position and frequency of both shared and de novo mtSNVs, *C9ORF72*-mutant hiPSCs formed a distinct cluster from their isogenic healthy counterparts. This indicates that the shared mtSNVs likely occurred shortly after the CRISPR correction going through clonal expansion. On the other hand, the dendrogram including solely de novo mutations showed no clustering, implying that these mtSNVs occurred at a much later stage. These expected findings endorse the robustness of our technical approach while indicating likely differences in the mtSNVs between the final cell lines (Fig. 2K).

### Selection against shared mtSNVs during organoid corticogenesis

**Fig. 3.**
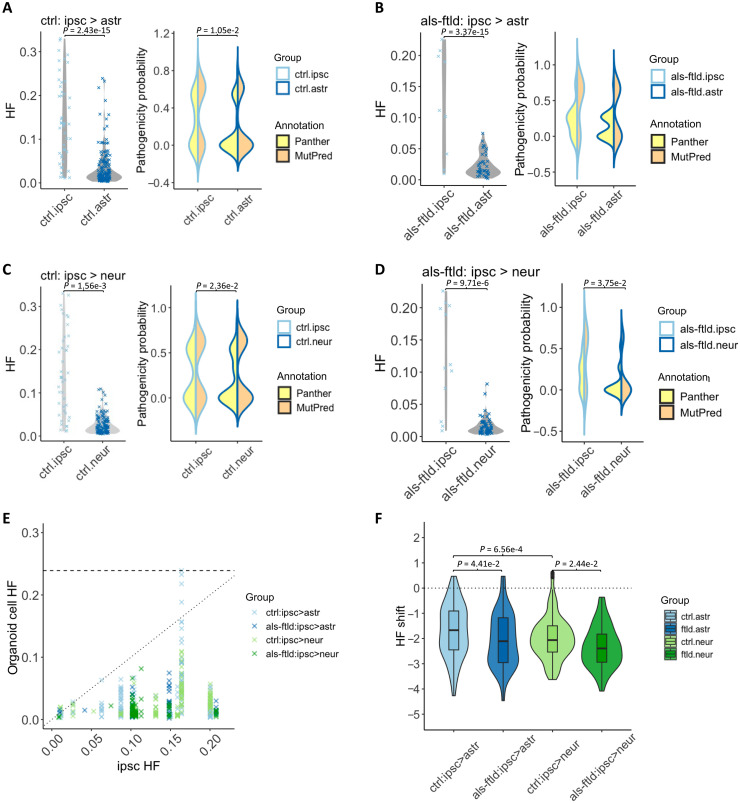
Shared mtSNVs during organoid corticogenesis. (**A**) Distribution of HF and pathogenicity scores (based on Panther and MutPred predictions) of mtSNVs detected both in hiPSCs and their differentiated astroglia in control (Wilcoxon test of distribution of HF: ctrl.ipsc versus ctrl.astr, *P* = 2.43 × 10^−5^; Wilcoxon test of MutPred score: ctrl.ipsc versus ctrl.astr, *P* = 1.05 × 10^−2^). (**B**) Distribution of HF and pathogenicity score of mtSNVs detected both in hiPSCs and their differentiated astroglia in *C9ORF72*-mutant organoids (Wilcoxon test of distribution of HF: als-ftld.ipsc versus als-ftld.astr, *P* = 3.37 × 10^−15^). (**C**) Distribution of HF and pathogenicity score of mtSNVs detected both in hiPSCs and their differentiated neurons in control (Wilcoxon test of distribution of HF: ctrl.ipsc versus ctrl.neur, *P* = 1.56 × 10^−3^; Wilcoxon test of MutPred score: ctrl.ipsc versus ctrl.neur, *P* = 2.36 × 10^−2^). (**D**) Distribution of HF and pathogenicity score of mtSNVs detected both in hiPSCs and their differentiated neurons in ALS-FTLD (Wilcoxon test of distribution of HF: als-ftld.ipsc versus als-ftld.neur, *P* = 9.71 × 10^−6^; Wilcoxon test of MutPred score: als-ftld.ipsc versus als-ftld.neur, *P* = 3.75 × 10^−2^). (**E**) HF of mtSNVs detected both in hiPSCs and organoid-derived cells (astroglia and neurons). (**F**) hiPSCs-to-astroglia or hiPSCs-to-neuron normalized heteroplasmy shift calculated, for mtSNVs detected both in hiPSCs and organoid-derived cells in ALS/FTLD and control (Kolmogorov-Smirnov test: ctrl.astr versus ctrl.neur, *P* = 6.56 × 10^−4^; ctrl.astr versus als-ftld.astr, *P* = 4.41 × 10^−2^; ctrl.neur versus als-ftld.neur, *P* = 2.44 × 10^−2^). ipsc, hiPSCs; astr, astroglia; neur, neurons; ctrl, control; als-ftld, ALS-FTLD.

Despite the increased mutational burden in the organoid-derived cells, the mtSNV HF was significantly lower in astroglia and neurons derived from both control hiPSCs (ctrl.ipsc versus ctrl.astr and ctrl.ipsc versus ctrl.neur, *P* < 0.05; Fig. 3, A and C, and fig. S4) and *C9ORF72*-mutant hiPSCs (als-ftld.ipsc versus als-ftld.astr and als-ftld.ipsc versus als-ftld.neur, *P* < 0.05; Fig. 3, B and D, and fig. S4). In keeping with Panther scores, MutPred predictions indicated a greater proportion of pathogenic mtSNVs in hiPSCs compared to organoid-derived differentiated cells, most notably for neurons in both control and *C9ORF72*-mutant organoids (ctrl.ipsc versus ctrl.astr, ctrl.ipsc versus ctrl.neur, and als-ftld.ipsc versus als-ftld.neur, *P* < 0.05; Fig. 3, A to D, and fig. S4). This body of evidence collectively implies that a significant number of mtSNVs present in hiPSCs were negatively selected against during the formation and maturation of cerebral organoids.

To explore this further, we studied the change of heteroplasmy values for specific mtSNVs between hiPSCs and their matched organoids. Calculating the normalized heteroplasmic shift controls for differences in the HF of individual mtSNVs within hiPSCs which can influence the absolute heteroplasmy shift (aHS) (*11*). In keeping with the earlier analysis, there was a significant negative aHS during both control and *C9ORF72*-mutant organoid formation. The selection was more pronounced in neurons than in astroglia in control organoids and more pronounced in both astroglia and neurons in *C9ORF72*-mutant organoids compared to the isogenic control organoid (ctrl.astr versus ctrl.neur, ctrl.astr versus als-ftld.astr, and ctrl.neur versus als-ftld.neur, *P* < 0.05; Fig. 3, E and F).

### Likely de novo mtSNVs

**Fig. 4.**
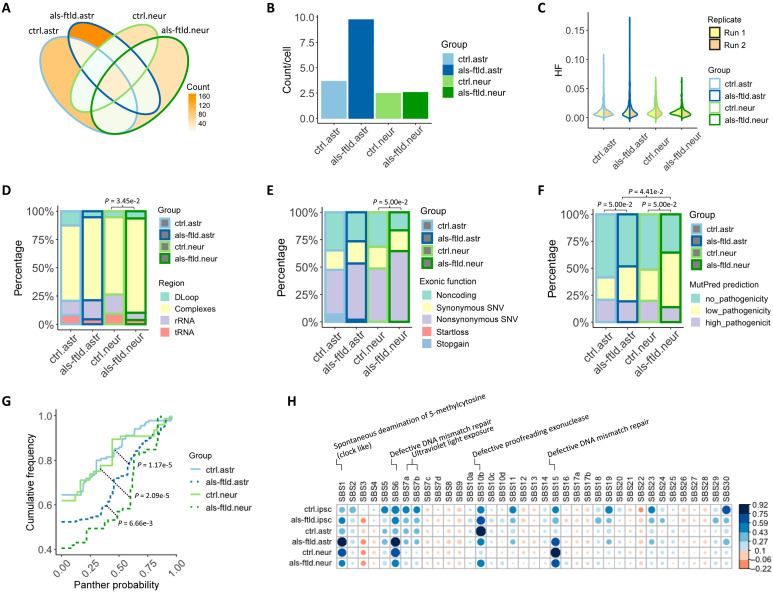
Likely de novo mtSNVs. (**A**) Count of mtSNVs detected in individual disease (ALS/FTLD and control) and cell type (astroglia and neurons) or intersected between groups. (**B**) Averaged count of de novo mtSNVs per cell in each group. (**C**) Distribution of HF of mtSNVs detected in each group. (**D** to **F**) Percentage of mtSNVs in each genomic region (Fisher’s exact test: ctrl.neur versus als-ftld.neur, *P* = 3.45 × 10^−2^), exonic function (Fisher’s exact test: ctrl.neur versus als-ftld.neur, *P* = 5.00 × 10^−2^), and MutPred prediction category (Fisher’s exact test: ctrl.astr versus als-ftld.astr, *P* = 5.00 × 10^−2^; ctrl.neur versus als-ftld.neur, *P* = 5.00 × 10^−2^; als-ftld.astr versus als-ftld.neur, *P* = 4.41 × 10^−2^) in each group. (**G**) Cumulative frequency of Panther probability of mtSNVs detected in each group (Kolmogorov-Smirnov test: ctrl.astr versus als-ftld.astr, *P* = 1.17 × 10^−5^; ctrl.neur versus als-ftld.neur, *P* = 2.09 × 10^−5^). (**H**) Correlation between trinucleotide mutational signatures observed in the current study with the 30 annotated cancer signatures (see Materials and Methods). The gradients of circles correspond to correlation *R*^2^ values, and the sizes of circles correspond to the *P* values (larger circle with lower *P* values). rRNA, ribosomal RNA; tRNA, transfer RNA; ipsc, hiPSCs; astr, astroglia; neur, neurons; ctrl, control; als-ftld, ALS-FTLD.

A greater number of likely de novo mtSNVs were seen per cell in astroglia from *C9ORF72*-mutant organoids than in neurons and in both cell types isolated from isogenic control organoids (Fig. 4, A to C). A higher proportion of mtSNVs were localized in mtDNA regions coding for respiratory chain complexes in *C9ORF72*-mutant organoids than isogenic controls (ctrl.neur versus als-ftld.neur, *P* < 0.05; Fig. 4D) and in protein-coding regions overall (ctrl.neur versus als-ftld.neur, *P* = 0.05; Fig. 4E). These mutations were predicted to be pathogenic by MutPred (ctrl.astr versus als-ftld.astr, ctrl.neur versus als-ftld.neur, and als-ftld.astr versus als-ftld.neur, *P* < 0.05; Fig. 4F). Supporting the marginal *P* values for this trend, the Panther probability of mtSNVs was higher in ALS/FTLD for both astroglia and neurons compared to healthy controls (ctrl.astr versus als-ftld.astr and ctrl.neur versus als-ftld.neur, *P* < 0.05; Fig. 4G and fig. S5).

We then determined the trinucleotide mutational signature (*12*) of the mtSNVs seen in hiPSCs and the likely de novo mtSNVs seen in astroglia and neurons. The mtSNV signature of hiPSCs correlated strongly with defective proofreading exonuclease (SBS10b) and ultraviolet light exposure (SBS7a and SBS7b), consistent with their proliferative nature and fibroblast origin. In contrast, the mtSNV mutation signature of neurons was more strongly associated with defective DNA mismatch repair (SBS6 and SBS15) and spontaneous deamination of 5-methylcytosine (clock-like signature), suggesting a divergence from hiPSCs. Compared to control astroglia, *C9ORF72*-mutant astroglia exhibited a higher prevalence of the clock-like signature and defective DNA mismatch repair, mirroring more of the neuronal mutational signature (Fig. 4H).

### Clonal origins of mtSNVs in cerebral organoids

**Fig. 5.**
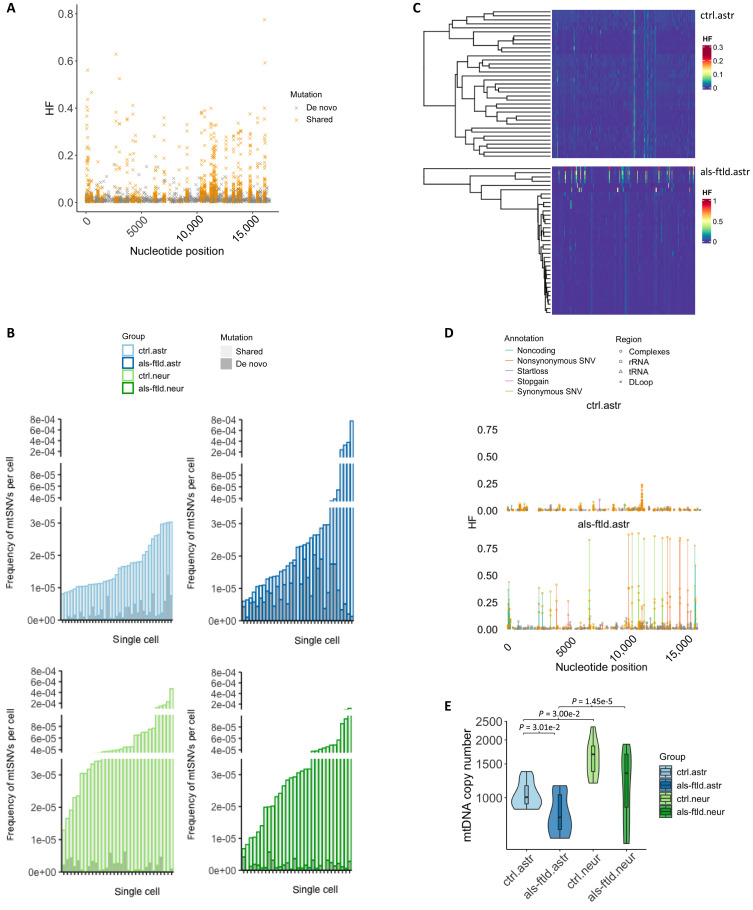
Clonal origins of mtSNVs in cerebral organoids. (**A**) Distribution of HF of shared or likely de novo mtSNVs. (**B**) The mutation frequency per cell in each group. Each bar represents a cell, color coded by the origin of mtSNVs (shared or likely de novo). (**C**) Identification of clonal subsets of astroglia in control or ALS/FTLD, based on mtSNVs (columns) sorted by unsupervised clustering. Color bar indicates the level of HF of each mtSNV. (**D**) Distribution of mtSNVs along mitochondrial genome. Each dot represents a mtSNV and each line a nucleotide position color coded by exonic function. (**E**) mtDNA copy number (CN) per cell detected by ddPCR in each group (one-way ANOVA with post hoc Tukey test: ctrl.astr versus ctrl.neur, *P* = 3.00 × 10^−2^; als-ftld.astr versus als-ftld.neur, *P* = 1.45 × 10^−5^; ctrl.astr versus als-ftld astr, *P* = 3.01 × 10^−2^). astr, astroglia; neur, neurons; ctrl, control; als-ftld, ALS-FTLD.

Next, we analyzed the HF and clonal distribution of mtSNVs in the cerebral organoids. As expected, variants found in more than one organoid cell type or in the hiPSCs (shared mtSNVs) had the highest HF, and variants only found in one cell type (de novo) had low HF (Fig. 5A). The higher HF of shared mtSNVs in some *C9ORF72*-mutant organoid-derived astroglia was also apparent in this analysis, with the low HF de novo mtSNVs in keeping with their more recent origin (Fig. 5B and figs. S6 to S8). mtDNA lineage tracing showed that the high HF mtSNVs in *C9ORF72*-mutant astroglia defined a distinct clade, which was not seen in control astroglia (Fig. 5C). Many of the high HF mtSNVs detected in *C9ORF72*-mutant astroglia located in complexes and ribosomal RNA coding regions, exceeding the conventional pathogenicity threshold frequency (HF > 0.6) (Fig. 5D and tables S5 and S6) (*13*). These findings endorse previous observations that cell lineage and cell type–specific mtSNVs potentially affect function in different subclones derived from hiPSCs.

Last, for completeness, we measured the single-cell mtDNA content by digital droplet polymerase chain reaction (ddPCR) in each group. Neurons contained a higher mtDNA copy number (CN) than astroglia, but CN was lower in *C9ORF72*-mutant cells than in the isogenic control, particularly in *C9ORF72*-mutant astroglia (ctrl.astr versus ctrl.neur, als-ftld.astr versus als-ftld.neur, and ctrl.astr versus als-ftld astr, *P* < 0.05) (Fig. 5E).

## DISCUSSION

Here, we show the single-cell mtSNV accumulation landscape in 133-day-old human ALS-FTLD cerebral organoids. While the observed similarities to postmortem brain mtDNA bulk sequencing data (*6*) validated our in vitro model, the organoid system allowed the elucidation of cell type specificity and the origin of mtSNV burden. We found that *C9ORF72*-mutant astroglia contain the highest burden of likely de novo mtSNVs. The mtSNVs within ALS-FTLD astroglia were predicted to be more likely to affect mitochondrial function. Together, these findings support a pathogenic role for both inherited and developmentally acquired mtSNVs in *C9ORF72*-mutant astroglia, with developmentally acquired variants potentially contributing to the pathology, particularly when reaching high HF. The mtDNA content of individual cells (also called CN) was lower in astroglia than neurons, particularly in *C9ORF72*-mutant cells. mtDNA CN typically increases in the presence of deleterious mutations, buffering the adverse effects of a mutation in a compensatory response (*14*). The relative suppression of mtDNA CN in *C9ORF72*-mutant astroglia could further exacerbate any pathological effects.

De novo mutations in mtDNA may arise due to oxidative stress, neuroinflammation, or disrupted energy metabolism, leading to direct damage by reactive oxygen species or indirectly increasing polymerase errors during ongoing mtDNA replication. These mechanisms exacerbate mitochondrial dysfunction, contributing to disease progression in ALS-FTLD. The accumulation of such mutations further amplifies energy deficits and neuronal loss, intensifying the neurodegenerative processes underlying ALS-FTLD. In contrast, mtDNA mutations that emerge after the early hiPSC stage and are shared between astroglia and neurons are likely acquired at low heteroplasmy, evading selection pressures during organoid formation and maturation. These variants can subsequently achieve high HFs, particularly in ALS-FTLD astroglia, potentially making them more relevant to pathology. Notably, in contrast to single-nucleotide variants, no mtDNA rearrangements were detected in any of the cell lines based on deep sequencing data analyzed using MitoSAlt (*15*).

The role of astroglia in the pathogenesis of FTLD and ALS-FTLD is gaining momentum (*16*). Astroglia not only release trophic factors to support neuronal survival and express inflammatory cytokines, triggering cascades to safeguard synapses (*17*) but also engage in interactions with various cell types, contributing to the construction of the blood-brain barrier and facilitating nutrient transport to neurons (*18*, *19*). Glial cell dysfunction and their miscommunication with immune cells have been identified as playing a pivotal role in ALS-FTLD, especially in haploinsufficiency or repeat-associated toxicity models of *C9ORF72* pathogenesis (*20*–*22*). Mitochondrial dysfunction in glial cells has attracted therapeutic interest as it contributes to the disruption of cellular homeostasis in ALS-FTLD, encompassing alterations to metabolism, redox homeostasis, Ca^2+^ signaling, inflammation, and cell death (*23*). However, the extent to which glial mtDNA is exposed to secondary mutations underlying the observed mitochondrial dysfunction has been unexplored (*6*, *24*). Our observations indicate that astroglia accumulate a higher burden of mtSNVs with a higher HF than neurons. These variants are more likely to compromise astroglial oxidative phosphorylation and its downstream consequences, thereby contributing to the pathogenesis of ALS-FTLD. In alignment with our findings, a recent study demonstrated mitochondrial malfunction and atrophy in aged astroglia, as opposed to neurons, in the human cerebral cortex (*25*).

Overall, our work has identified cell type and lineage-specific differences in the burden of mtDNA mutations which can influence oxidative phosphorylation and adenosine triphosphate synthesis, complementing previous clinical and pathophysiological studies of ALS-FTLD. From a technical perspective, our findings also provide an explanation for the lack of reproducibility and the generalizability of results from the analysis of individual cell lines derived from hiPSCs: The lineage and cell type–specific mtSNVs we have detected are likely to have different downstream consequences on different subclonal lines, potentially leading to misleading results and inconsistencies unless carefully controlled for. Given the clonal origin of some high heteroplasmic variants affecting some cell clades but not others, mtSNVs provide a plausible explanation for the regional pathology in ALS-FTLD, opening avenues for mitochondrially targeted therapies.

## MATERIALS AND METHODS

### Experimental design

hiPSCs used for single-cell sequencing were derived from two patients with ALS-FTLD, both carrying pathogenic HREs in the *C9ORF72* gene. This included mutant lines with approximately 800 repeats (als-ftld.a: CS29iALS-C9nxx) and 70 repeats (als-ftld.b: CS30iALS-C9nxx). Both cell lines exhibited a consistent disease phenotype characterized by frontotemporal dementia and/or ALS 1 (NCIt: C168756) and ALS (ORDO: Orphanet_803) (*26*). A control hiPSC line (ctrl.a) was generated by CRISPR-Cas9–mediated excision of the hexanucleotide repeat in the *C9ORF72* gene from the patient-derived als-ftld.a line, while another control line (ctrl.b) was obtained from a healthy donor for comparative analysis (table S1). For biological replication, brain organoid slice cultures were independently differentiated and grown separately from distinct 50-day-old brain organoids for each of the four lines as previously described (*8*) and cultured for a further 83 days (133 days in total). Specifically, we processed seven organoid slice cultures (derived from three separate organoids) for the ctrl.a and als-ftld.a lines, eight slices (from three separate organoids) for the ctrl.b line, and seven slices (from two distinct organoids) for the als-ftld.b line. The HRE sizes in both mutant and control lines were experimentally validated in our recent study using Southern blotting and PCR (*8*).

### Generation and characterization of isogenic corrected hiPSCs

hiPSCs were generated by reprogramming ALS/FTLD and control patient-derived fibroblasts using non-integrating episomal plasmids at the Cedars-Sinai Stem Cell Core (USA) and the European Bank for Induced Pluripotent Stem Cells (EBiSC). *C9ORF72* isogenic control clones were generated from *C9ORF72* hiPSCs using the CRISPR-Cas9 complex targeting the regions immediately 5′ and 3′ of the repeat expansion, which we refer to as ctrl.a line (CS29iALS-C9n1.ISOxx) and is a derivative of the als-ftld.a line (CS29iALS-C9nxx). The hiPSC lines were characterized by multiple strategies, including karyotyping (no nuclear chromosomal duplications were detected), pluripotency (SOX2 expression and their ability to produce organoids with diverse neural cell populations), and the rescue of pathological hallmarks by comparing the als-ftld.a line (CS29iALS-C9nxx) to its isogenic mutation corrected pair, the ctrl.a line (CS29iALS-C9n1.ISOxx). The results of basic and pathological characterizations using the same hiPSCs have been published in our recent work (*8*) and by EBiSC and the Cedars-Sinai Core where the lines were obtained from.

### Single-cell astroglia and neuron sorting

For FACS-based cell separation of control and ALS-FTLD brain organoid slice cultures, the expressions of astroglia and neuron-specific markers were validated by meta-analysis of previously published scRNA-seq data (*8*) using the same pipelines. To demonstrate gene expression levels across the cell populations, feature plots were produced in Seurat 4.0.1. Astroglia and neuron-specific markers were visualized in 12-mm–thick cryostat sections of 4% paraformaldehyde-fixed brain organoids by standard immunohistochemical methods (*8*) using cell type–specific markers indicated in table S2. For illustration, immunofluorescence images were taken by the Leica DM6000 microscope, and camera settings were kept the same while collecting images. The recommended guidelines were used for presentation in the figures. For cell sorting, organoids were washed in 1× Dulbecco’s Phosphate Buffered Saline (dPBS) (Sigma-Aldrich, D8537), before being placed into gentleMACS^T^ C tubes (Miltenyi, 130-093-237) in 2 ml of TrypLE (Thermo Fisher Scientific, 12605010) for tissue dissociation. Single-cell suspension was achieved using the Adult Brain Dissociation Kit (ABDK) program of the gentleMACSTM Octo Dissociator (Miltenyi, 130-096-427). After dissociation, the solution was diluted in dPBS containing deoxyribonuclease (0.5 mg ml^−1^; Sigma-Aldrich, 11284932001) before a 5-min centrifugation at 300*g*. The cell pellet was resuspended and filtered using a 70-μm strainer (Miltenyi, 130-098-462) and centrifuged again. Dissociated single cells were then stained with a cell viability dye [Zombie-near infrared (NIR)] and labeled with astroglia (HEPACAM)– or neuron (L1CAM)–specific markers (table S2) before FACS into 96-well plates using the following protocol. The pellet was resuspended in dPBS containing 0.05% bovine serum albumin (BSA; Sigma-Aldrich, A9418) and the primary antibodies and incubated for 1 hour on a shaker at 37°C. The samples were then washed in 0.5% BSA dPBS and centrifuged for 5 min at 300*g*, before resuspended in the secondary antibody solution and incubated for another hour on the shaker at 37°C. After the final wash, cells were resuspended at 10^6^ cells ml^−1^ and sorted using a BD FACSAria Flow Cytometer. Automatic normalization was applied using individually stained samples and a negative control. The gating was determined using an isotype control (mouse immunoglobulin G1 antibody; fig. S1). Cells were filtered for doublets and debris, and only HEPACAM-positive astroglia or L1CAM-positive neurons were retained. Postsorting, the plates underwent centrifugation at 2000*g* for 1 min at 4°C, followed by flash freezing on dry ice and subsequent storage at −80°C for long-term preservation.

### Single-cell mtDNA sequencing

FACS-sorted single cells were lysed under alkaline conditions on ice to minimize lysis-induced artifacts (*10*) before whole mtDNA amplification using long-range PCR (detailed in table S3). Amplicons were sequenced at high depth using Illumina Nextera XT reagents and Illumina MiSeq v3 chemistry. Each DNA sample underwent duplicate sequencing, including independent stages of PCR amplification, library preparation, and sequencing.

### Bioinformatic analysis and annotation of mtDNA variants

Sequencing reads were aligned to the human reference genome (hg19 and rCRS NC_012920.1) using MToolBox (v1.1) incorporating a two-stage mapping approach to filter out technical artifacts due to alignment against nuclear DNA of mitochondrial origin (NuMTs) (*27*). The probabilistic model RePlow (v1.1.0) was used on library-level replicates to enhance stringency in identifying artifacts during the calling of low-heteroplasmy (HF) variants (HF > 0.005) (*9*). mtDNA variants located in homopolymeric regions were excluded from analysis due to the known high frequency of artifacts. Annotation and pathogenicity prediction of mtSNVs were conducted using mtoolnote (v0.2.0). Mutational signatures were analyzed based on single-site pyrimidine substitutions adjacent to 5′ and 3′ bases, forming 96 trinucleotide signatures. Substitution rates for each trinucleotide context on the mtDNA light (L) and heavy (H) strands were normalized against the frequency of the trinucleotide context in the mtDNA reference genome (rCRS), and comparisons were made to the 30 identified nuclear genomic signatures (*12*). An independent variant caller mgatk (v0.6.6) was applied to cross-validate the current analysis, generating single-cell lineage maps based on their variants and HF (*28*).

### scRNA-seq pipeline and analysis

The scRNA-seq data were processed as previously reported (*8*) using CellRanger (v3.1) and Seurat (v4.0.1), filtering for quality and viable cells (expressing 200 to 5000 genes and <25% mitochondrial genes). Clustering revealed cell types based on marker expression. Batch correction was conducted, and differential expression analysis identified genes altered in *C9ORF72*-mutant organoid compared to controls.

### Bulk DNA extraction and sequencing

hiPSC, astroglia, and neuron pellets from both control and ALS-FTLD groups were flash-frozen in liquid nitrogen and stored at −80°C. Genomic DNA was extracted using the Monarch genomic DNA extraction & purification system [New England Biolabs (NEB), T3010S]. The quality of nuclear DNA obtained from the cell pellets was assessed using genomic DNA ScreenTape assays (Agilent, 5067-5365) and screened on the basis of DNA integrity numbers. Next generation sequencing (NGS) libraries were prepared as described above. MtDNA haplogroups were determined from the sequence data using MToolBox (*27*) and compared them against reference databases including PhyloTree (*29*), confirming all samples derived from the same parental hiPSC assigned to the same haplogroup H3am.

### Measurement of mtDNA CN

Single-cell mtDNA CN was measured by ddPCR as described (*30*). Primer and probe sequences used are provided (table S4).

### Statistical analysis

To assess the congruence between the two independent sequencing runs (run 1 and run 2), we used a coefficient of determination (*R*^2^) analysis, fitting a linear regression model. For comparing mtDNA CNs and mutation counts across different groups, a one-way analysis of variance (ANOVA) with post hoc Tukey test was used. The distribution of the HF of mtSNVs across various genomic subregions, as well as within pathogenicity subgroups, was compared using the Wilcoxon rank-sum test. This nonparametric test was also applied to assess differences in Panther, MutPred, and PolyPhen-2 probability scores of mtSNVs between various disease groups and cell types. To examine the cumulative frequency of heteroplasmic mtSNVs across different disease conditions and cell types, the two-sample Kolmogorov-Smirnov test was used. The same statistical approach was used to compare shifts in HF between groups (*11*). Fisher’s exact test was conducted to compare the proportion of mtSNVs attributable to different functional groups annotated by mtoolnote, different pathogenic categories predicted by MutPred, and across mitochondrial genomic regions. To further delve into the mtSNV signature profiles across different groups, the frequency of 96 trinucleotide mutations was estimated individually. To explore the underlying mechanisms of mutagenesis, the signature profiles of mtDNA substitutions from various groups (ctrl.ipsc, als-ftld.ipsc, ctrl.astr, als-ftld.astr, ctrl.neur, and als-ftld.neur) were correlated with 30 cancer-specific mutational signatures in nuclear DNA, quantified using Pearson’s correlation coefficients (*12*, *31*). Hierarchical clustering analysis was done applying the hclust module in R (v3.4).
