# Supplementary material for: Origin and cell type specificity of mitochondrial DNA mutations in C9ORF72 ALS-FTLD human brain organoids
Source: Sci Adv. 2025 Mar 7;11(10):eadr0690. doi: 10.1126/sciadv.adr0690 (PMC11887808; doi:10.1126/sciadv.adr0690)
Supplement: Supplementary file 1 — Figs. S1 to S8 Legends for tables S1 to S6 [file sciadv.adr0690_sm.pdf]

Science Advances
AAAS

Supplementary Materials for

Origin and cell type specificity of mitochondrial DNA mutations in *C9ORF72* ALS-FTLD human brain organoids

Yu Nie *et al.*

Corresponding author: Andras Lakatos, al291@cam.ac.uk; Patrick F. Chinnery, pfc25@cam.ac.uk

Sci. Adv. **11**, eadr0690 (2025)
DOI: 10.1126/sciadv.adr0690

The PDF file includes:

Figs. S1 to S8
Legends for tables S1 to S6

Other Supplementary Material for this manuscript includes the following:

Tables S1 to S6

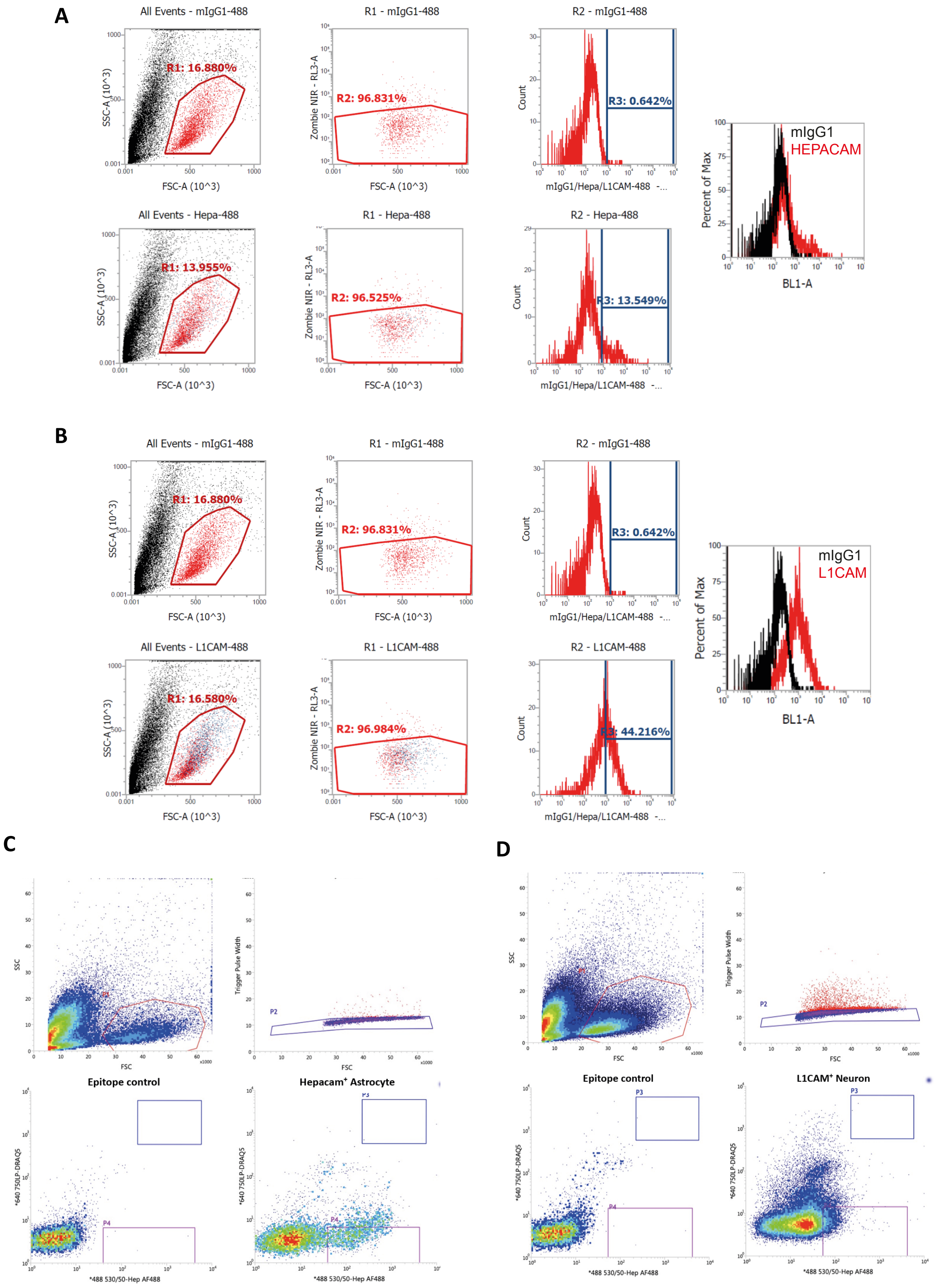
A
All Events - mIgG1-488
R1: 16.880%
SSC-A (10^3)
FSC-A (10^3)
R1 - mIgG1-488
R2: 96.831%
Zombie NIR - RL3-A
FSC-A (10^3)
R2 - mIgG1-488
R3: 0.642%
Count
mIgG1/Hepa/L1CAM-488 -...
All Events - Hepa-488
R1: 13.955%
SSC-A (10^3)
FSC-A (10^3)
R1 - Hepa-488
R2: 96.525%
Zombie NIR - RL3-A
FSC-A (10^3)
R2 - Hepa-488
R3: 13.549%
Count
mIgG1/Hepa/L1CAM-488 -...
mIgG1
HEPACAM
Percent of Max
BL1-A
B
All Events - mIgG1-488
R1: 16.880%
SSC-A (10^3)
FSC-A (10^3)
R1 - mIgG1-488
R2: 96.831%
Zombie NIR - RL3-A
FSC-A (10^3)
R2 - mIgG1-488
R3: 0.642%
Count
mIgG1/Hepa/L1CAM-488 -...
All Events - L1CAM-488
R1: 16.580%
SSC-A (10^3)
FSC-A (10^3)
R1 - L1CAM-488
R2: 96.984%
Zombie NIR - RL3-A
FSC-A (10^3)
R2 - L1CAM-488
R3: 44.216%
Count
mIgG1/Hepa/L1CAM-488 -...
mIgG1
L1CAM
Percent of Max
BL1-A
C
SSC
FSC
Trigger Pulse Width
P2
FSC
Epitope control
Hepacam+ Astrocyte
*640 750LP-DRAQ5
P4
*488 530/50-Hep AF488
D
SSC
FSC
Trigger Pulse Width
P2
FSC
Epitope control
L1CAM+ Neuron
P3
P4
*640 750LP-DRAQ5
*488 530/50-Hep AF488

Fig. S1. A. Cytometric analysis of HEPACAM immunoreactivity for astroglia selection. B. Cytometric analysis of L1CAM immunoreactivity for neuron selection. C and D. Gating strategy for astrocyte (C) and neuron (D) cell sorting. P4 represents the window for positively sorted cells.

A

B

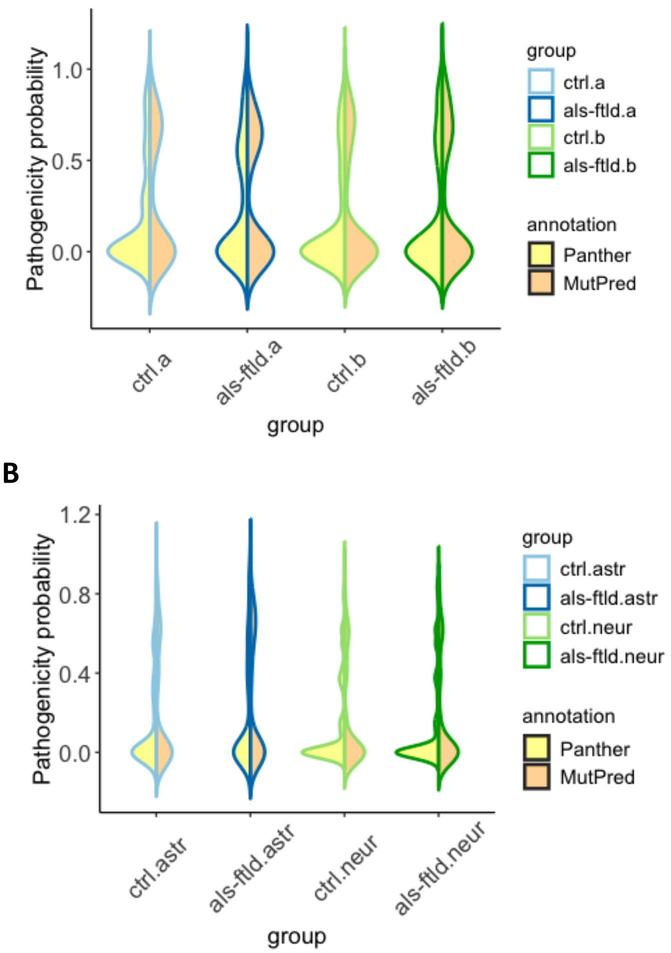

Fig. S2. Pathogenic probability predicted by Panther and MutPred, of mtSNVs detected in hiPSCs (Wilcoxon test: als-ftld.a vs ctrl.b, $p = 4.15e\text{-}2$) (A) and organoid derived astroglia and neurons (Wilcoxon test: ctrl.astr vs als-ftld.astr, $p = 2.02e\text{-}2$) (B).

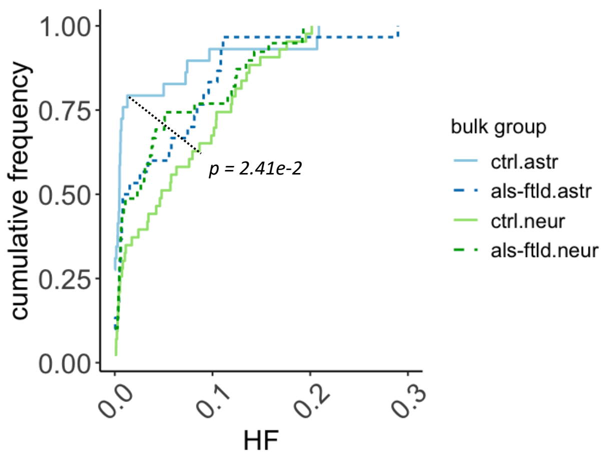

Fig. S3. Cumulative frequency of heteroplasmy fraction (HF) of mtSNVs detected in each group at bulk level.

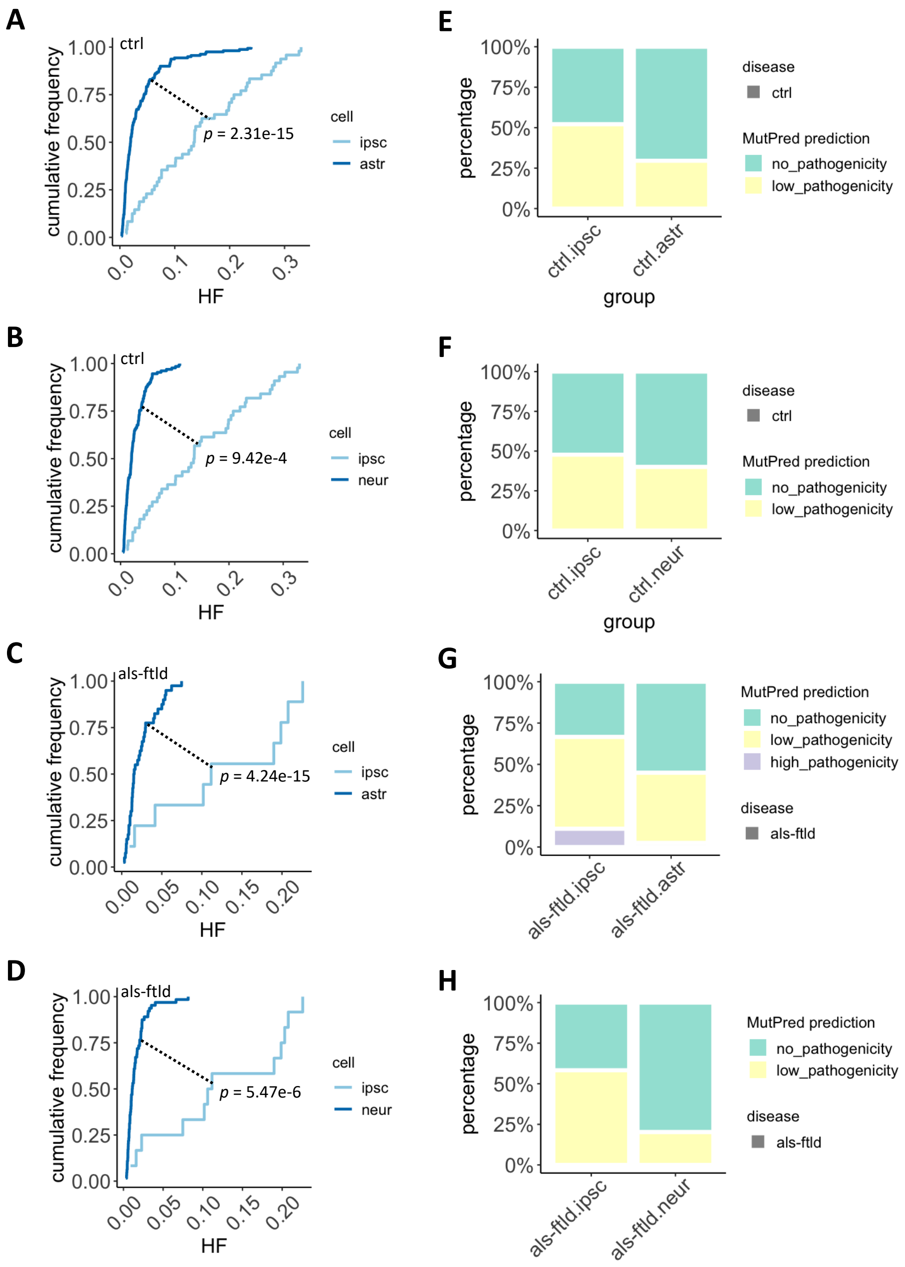

Fig. S4. A-D. Cumulative frequency of heteroplasmy fraction (HF) of mtSNVs detected in both hiPSCs and their derived organoid cells (astroglia and neurons). E-F. Percentage of shared mtSNVs in each MutPred prediction category.

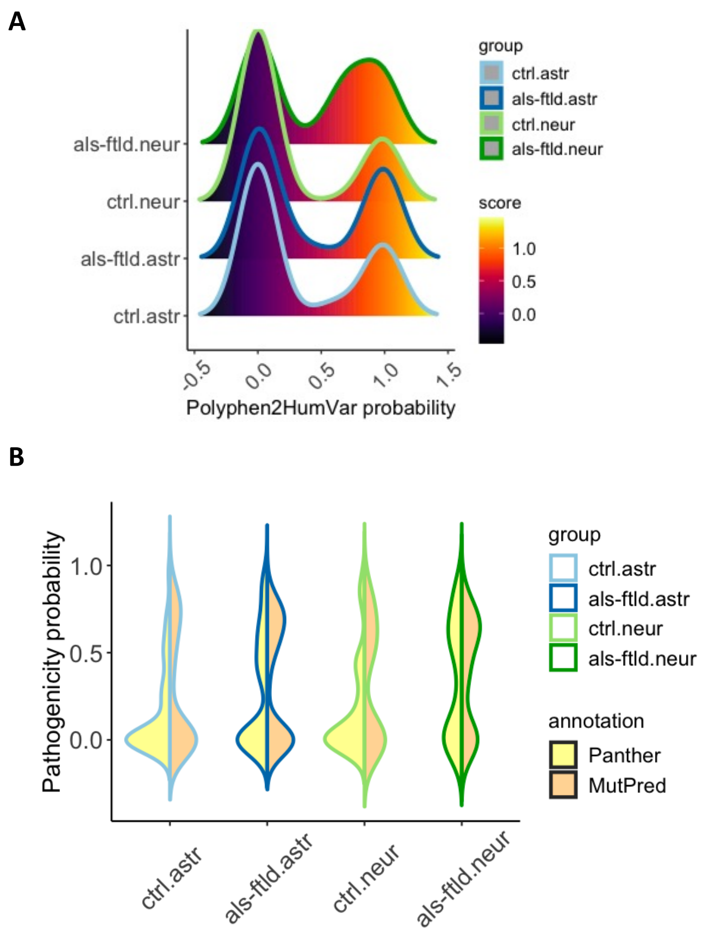

Fig. S5. Pathogenic probability predicted by PolyPhen-2, Panther and MutPred, of *de novo* mtSNVs detected in each group.

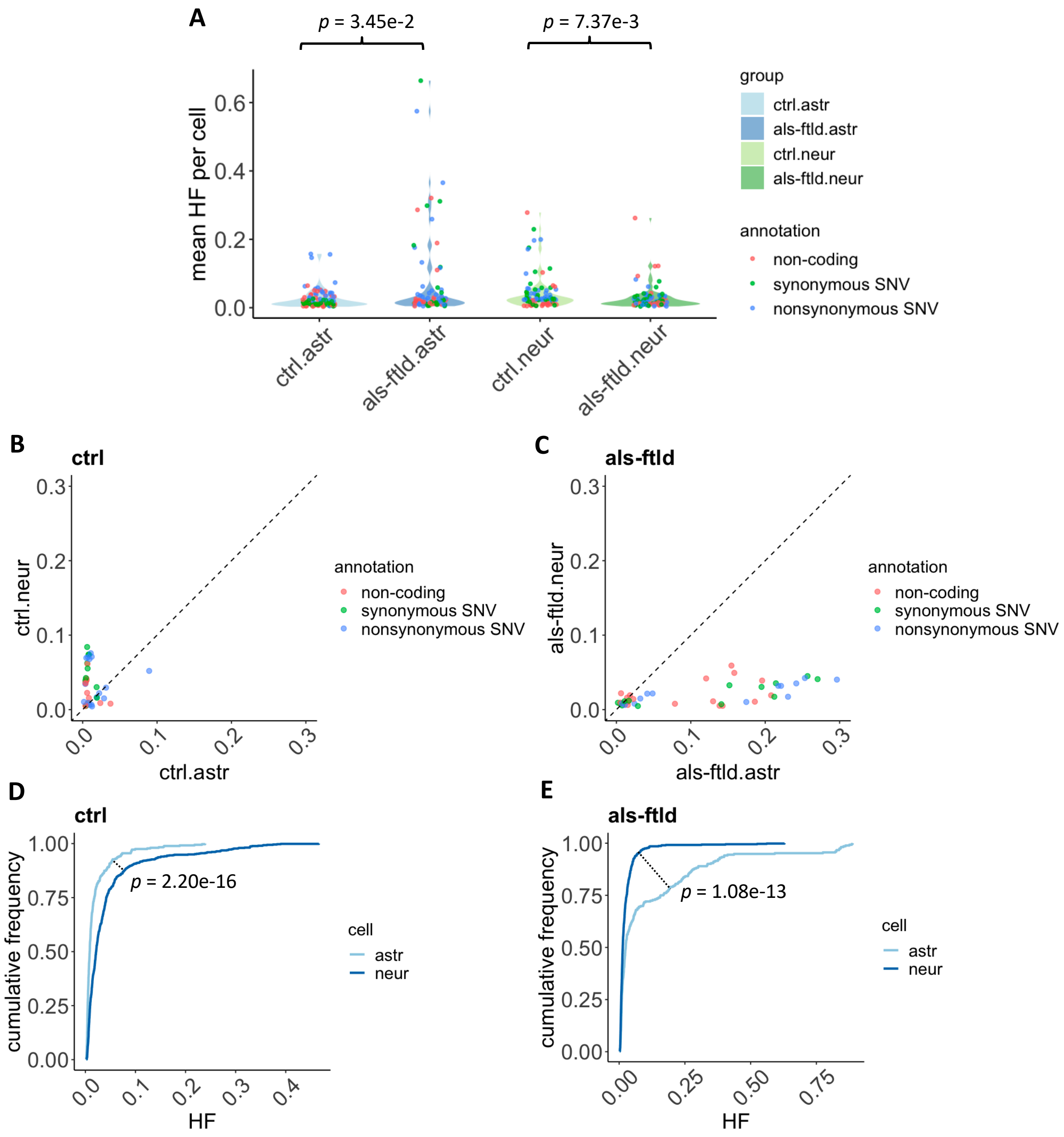

Fig. S6. A. Averaged heteroplasmy fraction (HF) per cell in each group. B-C. HF of mtSNVs detected both in astroglia and neurons, in control and ALS-FTLD. D-E. Cumulative frequency of HF of mtSNVs detected both in astroglia and neurons.

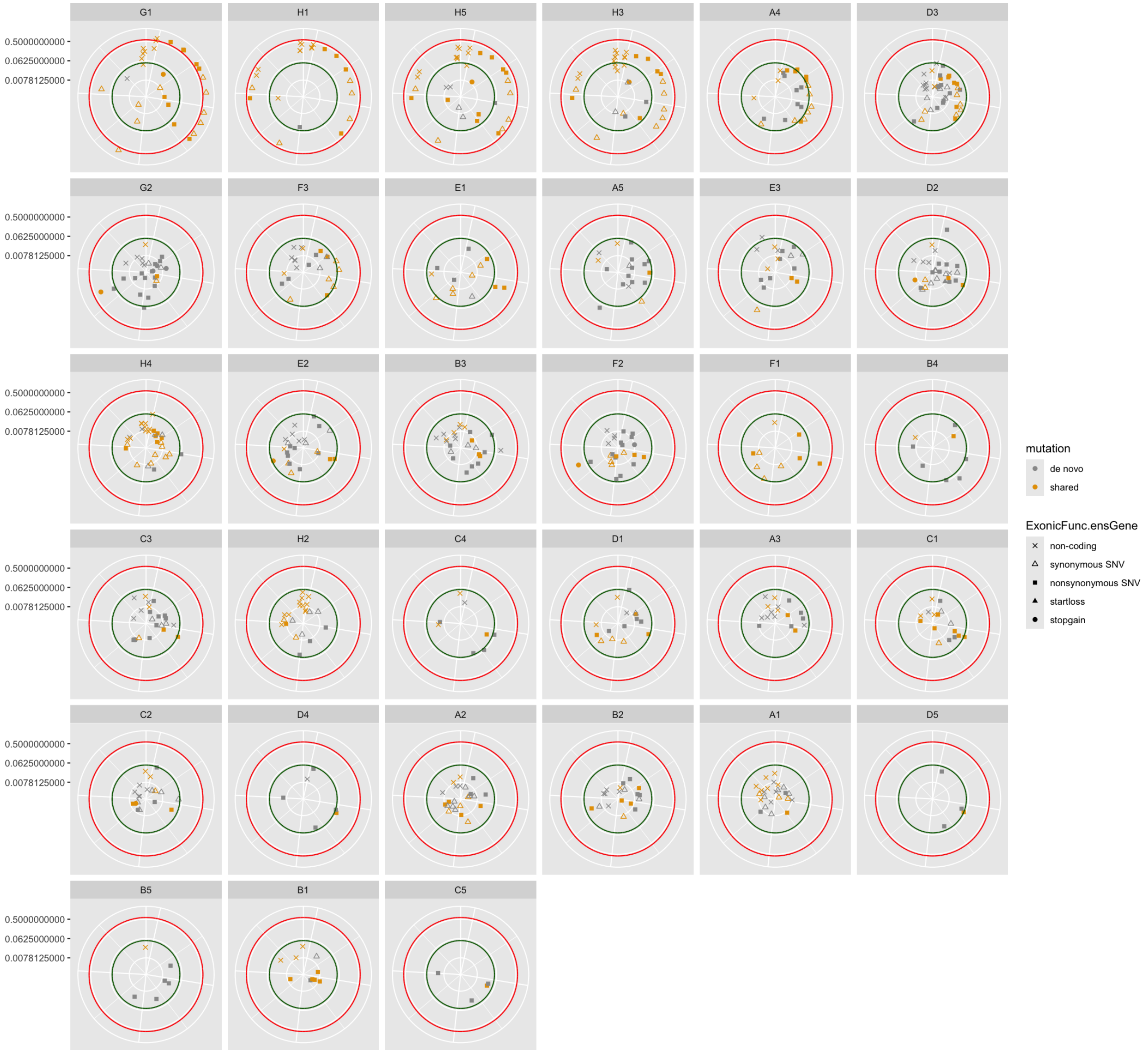
G1
H1
H5
H3
A4
D3
G2
F3
E1
A5
E3
D2
H4
E2
B3
F2
F1
B4
C3
H2
C4
D1
A3
C1
C2
D4
A2
B2
A1
D5
B5
B1
C5
0.5000000000
0.0625000000
0.0078125000
mutation
de novo
shared
ExonicFunc.ensGene
non-coding
synonymous SNV
nonsynonymous SNV
startloss
stopgain

Fig. S7. mtSNVs are distributed among individual astroglial cells in the ALS-FTLD group – part1. Each solar plot represents all detected mutations, including both *de novo* (gray) and shared mtSNVs (orange) within a single cell. The exonic function of each mutation is indicated by shape, as shown in the legend. The heteroplasmy frequency (HF) of mtSNVs is log2-transformed, with thresholds of 0.05 (5%) and 0.6 (60%) marked by green and red lines, respectively.

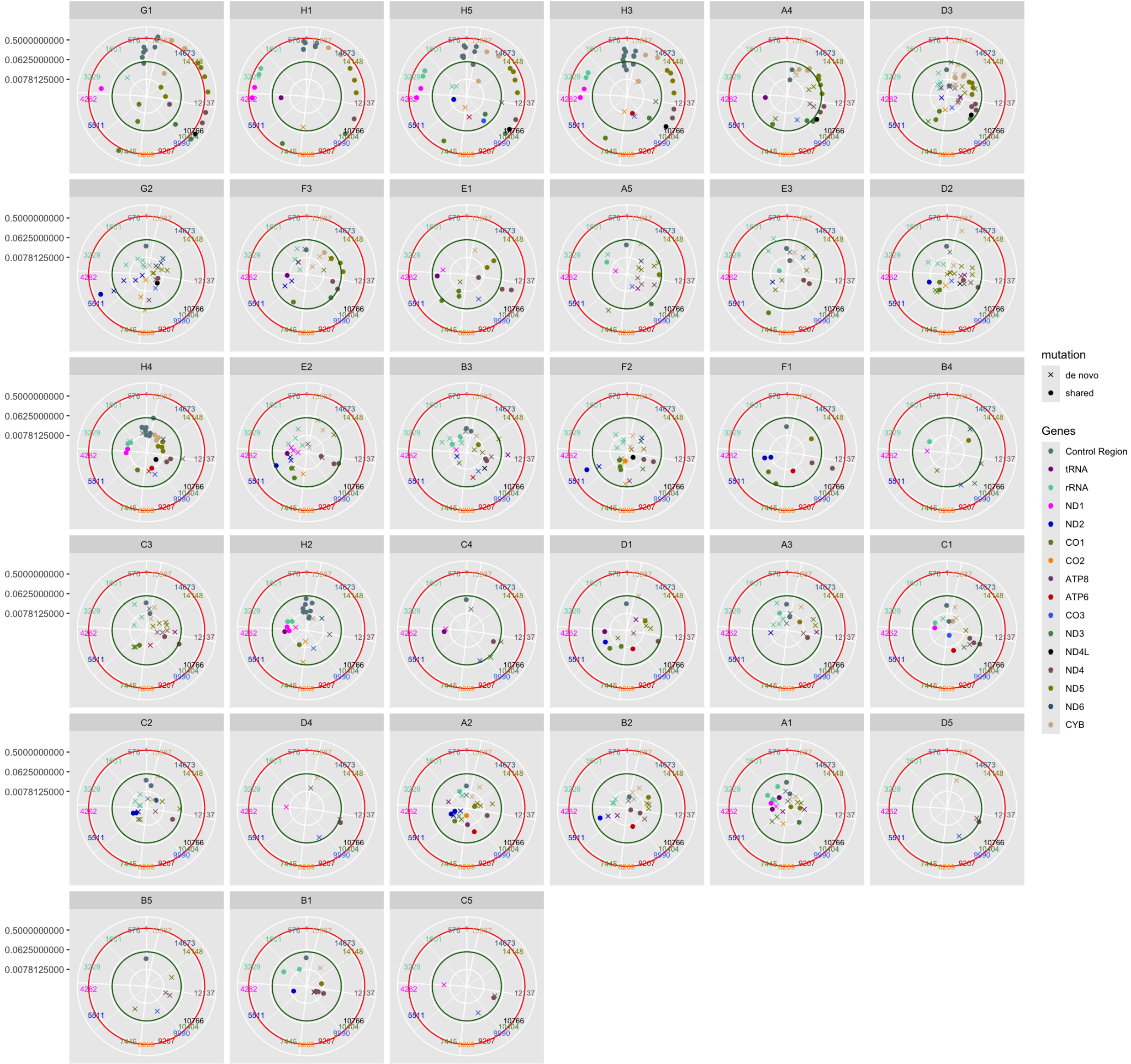

G1
H1
H5
H3
A4
D3
G2
F3
E1
A5
E3
D2
H4
E2
B3
F2
F1
B4
C3
H2
C4
D1
A3
C1
C2
D4
A2
B2
A1
D5
B5
B1
C5
0.5000000000
0.0625000000
0.0078125000
576
1601
3229
4262
5511
7445
9207
9990
10404
10766
12137
14148
14673
mutation
de novo
shared
Genes
Control Region
tRNA
rRNA
ND1
ND2
CO1
CO2
ATP8
ATP6
CO3
ND3
ND4L
ND4
ND5
ND6
CYB

Fig. S8. mtSNVs are distributed among individual astroglial cells in the ALS-FTLD group – part2. Each solar plot represents all mutations, across all mitochondrial genomic regions (colored coded), detected in a single cell. The origin of the mutation (*de novo* vs. shared mtSNVs) follows shape codes. The HF of mtSNVs is log2-transformed, with thresholds of 0.05 (5%) and 0.6 (60%) marked by green and red lines, respectively.

Table S1. hiPSC lines used for organoid generation [8] and single cell sequencing. *This line was generated from the ALS/FTLD.a using the CRISPR-Cas9 targeting the regions immediately 5' and 3' of the repeat expansion.

Table S2. Dyes and antibodies used for organoid derived single cell sorting.

Table S3. Primer pairs (forward and reverse) used for LR-PCR of mtDNA.

Table S4. Primers and probes used for ddPCR of mtDNA.

Table S5. The mutation annotation table of shared high-HF (HF above 0.6) mtSNVs observed in astroglial cells within the ALS-FTLD group-part1. The table provides a comprehensive summary of mitochondrial genome variants and their predicted functional impacts. Key columns include pos (position), ref (reference allele), and alt (alternate allele), describing the nucleotide change at a specific genomic location. Func.ensGene indicates the variant's functional classification within the gene, such as exonic or intronic, while Gene.ensGene specifies the Ensembl gene identifier associated with the mutation. When the variant is exonic, ExonicFunc.ensGene describes its impact at the protein level, such as a nonsynonymous single nucleotide variant (SNV) that changes the amino acid sequence. AAChange.ensGene provides a detailed annotation of the amino acid substitution, formatted as gene:transcript:exon change.

Table S6. The mutation annotation table of shared high-HF (HF above 0.6) mtSNVs observed in astroglial cells within the ALS-FTLD group-part2. The table includes several variant effect prediction scores. SNPsGO_Probability reflects the probability from SNPs&GO, a method predicting pathogenicity based on evolutionary information, gene ontology, and functional data. The PolyPhen-2 scores are divided into Polyphen2HumDiv_Prediction and Polyphen2HumVar_Prediction, each with a probability value. The HumDiv model assesses whether a mutation might impact protein structure and function, emphasizing high-confidence predictions for Mendelian variants (germline changes linked to disease), while the HumVar model is calibrated for common variants, providing sensitivity across a broader range of human genetic diversity; both models label variants as benign, possibly damaging, or probably damaging, with probabilities indicating prediction confidence. Conservation and pathogenicity metrics give additional insight, with NtVarH and AaVarH measuring nucleotide and amino acid conservation across species (higher scores reflect more conserved, potentially functional positions), and NtVarP and AaVarP estimating pathogenicity probability at nucleotide and amino acid levels, aiding in variant prioritization for further study. Clinvar provides ClinVar IDs for known variants, linking them to clinical annotations if available, thereby contextualizing each variant within known clinical databases and highlighting potential disease associations. Altogether, these annotations offer a multi-dimensional assessment of each variant's potential impact.
